# Ribonuclease H1-targeted R-loops in surface antigen gene expression sites can direct trypanosome immune evasion

**DOI:** 10.1371/journal.pgen.1007729

**Published:** 2018-12-13

**Authors:** Emma Briggs, Kathryn Crouch, Leandro Lemgruber, Craig Lapsley, Richard McCulloch

**Affiliations:** The Wellcome Centre for Molecular Parasitology, University of Glasgow, College of Medical, Veterinary and Life Sciences, Institute of Infection, Immunity and Inflammation, Glasgow, United Kingdom; University of Washington School of Medicine, UNITED STATES

## Abstract

Switching of the Variant Surface Glycoprotein (VSG) in *Trypanosoma brucei* provides a crucial host immune evasion strategy that is catalysed both by transcription and recombination reactions, each operating within specialised telomeric VSG expression sites (ES). VSG switching is likely triggered by events focused on the single actively transcribed ES, from a repertoire of around 15, but the nature of such events is unclear. Here we show that RNA-DNA hybrids, called R-loops, form preferentially within sequences termed the 70 bp repeats in the actively transcribed ES, but spread throughout the active and inactive ES, in the absence of RNase H1, which degrades R-loops. Loss of RNase H1 also leads to increased levels of VSG coat switching and replication-associated genome damage, some of which accumulates within the active ES. This work indicates VSG ES architecture elicits R-loop formation, and that these RNA-DNA hybrids connect *T*. *brucei* immune evasion by transcription and recombination.

## Author summary

All pathogens must survive eradication by the host immune response in order to continue infections and be passed on to a new host. Changes in the proteins expressed on the surface of the pathogen, or on the surface of the cells the pathogen infects, is a widely used strategy to escape immune elimination. Understanding how this survival strategy, termed antigenic variation, operates in any pathogen is critical, both to understand interaction between the pathogen and host, and disease progression. A key event in antigenic variation is the initiation of the change in expression of the surface protein gene, though how this occurs has been detailed in very few pathogens. Here we examine how changes in expression of the surface coat of the African trypanosome, which causes sleeping sickness disease, are initiated. We reveal that specialised nucleic acid structures, termed R-loops, form around the expressed trypanosome surface protein gene and increase in abundance after mutation of an enzyme that removes them, leading to increased changes in the surface coat in trypanosome cells that are dividing. We therefore shed light on the earliest acting events in trypanosome antigenic variation.

## Introduction

The genome provides the blueprint for life and is normally protected from rapid content change by high fidelity DNA replication and a range of repair pathways. However, strategies for elevated rates of genome variation have evolved, some of which are genome-wide, such as in developmental chromosome fragmentation in ciliates [1] and chromosome and gene copy number variation during *Leishmania* growth [2, 3]. More commonly, enhanced genome change is more localised and caused by deliberate lesion generation, such as during yeast mating type switching, which is induced by HO endonuclease-mediated cleavage in *Saccharomyces cerevisiae* [4] and locus-directed replication stalling in *Schizosaccharomyces pombe* [5]. Rearrangements to generate mature receptors and antibodies expressed by T and B cells [6] occur throughout mammalian immune genes and are generated by RAG endonuclease-catalysed DNA breaks [6] or transcription-linked base modification [7]. Reflecting the diversity in routes capable of initiating genome change, homologous recombination (HR), non-homologous end-joining and microhomology-mediated end-joining repair reactions have been implicated in the catalysis of these different reactions. Antigenic variation is a very widespread pathogen survival strategy, involving stochastic switches in surface antigens to thwart host adaptive immunity [8], and locus-directed gene rearrangement is a common route for the differing reactions used in bacteria, fungi and protists [9, 10]. Since antigenic variation impedes vaccination, it is important to understand the potentially pathogen-specific events that initiate surface antigen gene switching. However, only in the bacteria *Neisseria gonorrhoeae* is initiation well understood; here, HR catalyses movement of silent, non-functional *pilS* genes into a *pilE* expression locus via transcription-induced guanine quadruplex formation [11]. In no other pathogen has the initiation event(s) during antigenic variation been resolved in this detail.

Antigenic variation in *T*. *brucei* displays remarkable mechanistic complexity, since it involves both HR-directed rearrangement and transcriptional control of *VSG* genes. In any cell a single *VSG* is expressed by RNA Polymerase (Pol) I transcription of one of the multiple telomeric ES [12, 13]. In each ES the *VSG* is proximal to the telomere and is co-transcribed with multiple *ESAG*s (expression site associated genes) [14]. Invariably, the *VSG* and *ESAG*s are separated by stretches of 70 bp repeats, sequences also found upstream of thousands of further *VSG*s in the *T*. *brucei* genome [15–17]. To execute a VSG coat switch, *T*. *brucei* can use HR to move one of around 1,000 silent subtelomeric *VSGs* into the active VSG ES, displacing the resident *VSG*, or silence transcription from the active VSG ES and activate transcription from one of the silent VSG ES. Though a wide range of factors have been described that influence singular ES expression [12, 18–21] and execute VSG recombination [22], initiation of VSG switching remains poorly understood. Targeting yeast I-SceI endonuclease activity to the active VSG ES elicits *VSG* recombination [23, 24], but no endogenous ES-focused endonuclease has been described. Impaired telomere protection results in VSG ES breaks [25, 26] and critically short telomeres have been associated with increased VSG switching [27], but how such processes act in unperturbed cells is unknown. Finally, DNA replication mapping suggests that the active VSG ES, uniquely amongst these telomeric loci, is subject to early replication in mammal-infective (bloodstream form, BSF) parasite cells [28], but how this extrapolates to VSG switch initiation is unclear [29].

R-loops are stable RNA-DNA hybrids that form within the DNA helix, displacing single-stranded DNA. Though R-loops can arise from transcription, active roles are emerging in an range of genomic processes [30], including replication initiation [31, 32] and arrest [33], transcription activation and termination [30], telomere homeostasis [26, 34] and chromatin formation [35, 36]. In addition, R-loops can lead to genome instability and mutation [37–39]. To date, only one example of R-loop involvement in targeted genome rearrangement has been described: class switch recombination to alter the antibody type expressed by mature mammalian B lymphocytes [40–42]. Amongst a potentially wide range of activities described that prevent, reverse or interact with R-loops [30, 43, 44], RNase H enzymes function to degrade the RNA within the hybrids. In virtually every cellular organism, two distinct ribonuclease H enzymes, which are termed RNase H1 and RNase H2 in eukaryotes, have been described [45]. Here, we describe R-loop distribution in the VSG ES of both wildtype BSF *T*. *brucei* and in null mutants that lack a homologue of RNase H1. We show that R-loops accumulate throughout all VSG ES in the absence of the RNase H enzyme, indicating RNA-DNA hybrids form in these transcription sites and are normally resolved by removing the RNA. Loss of the RNase H results in elevated levels of replication-associated damage and leads to increased VSG coat switching, suggesting a model for the events that initiate antigenic variation in *T*. *brucei* and potential parallels with R-loop directed mammalian class switch recombination.

## Results

### *T*. *brucei* encodes a non-essential, nuclear RNase H1

**Fig 1:**
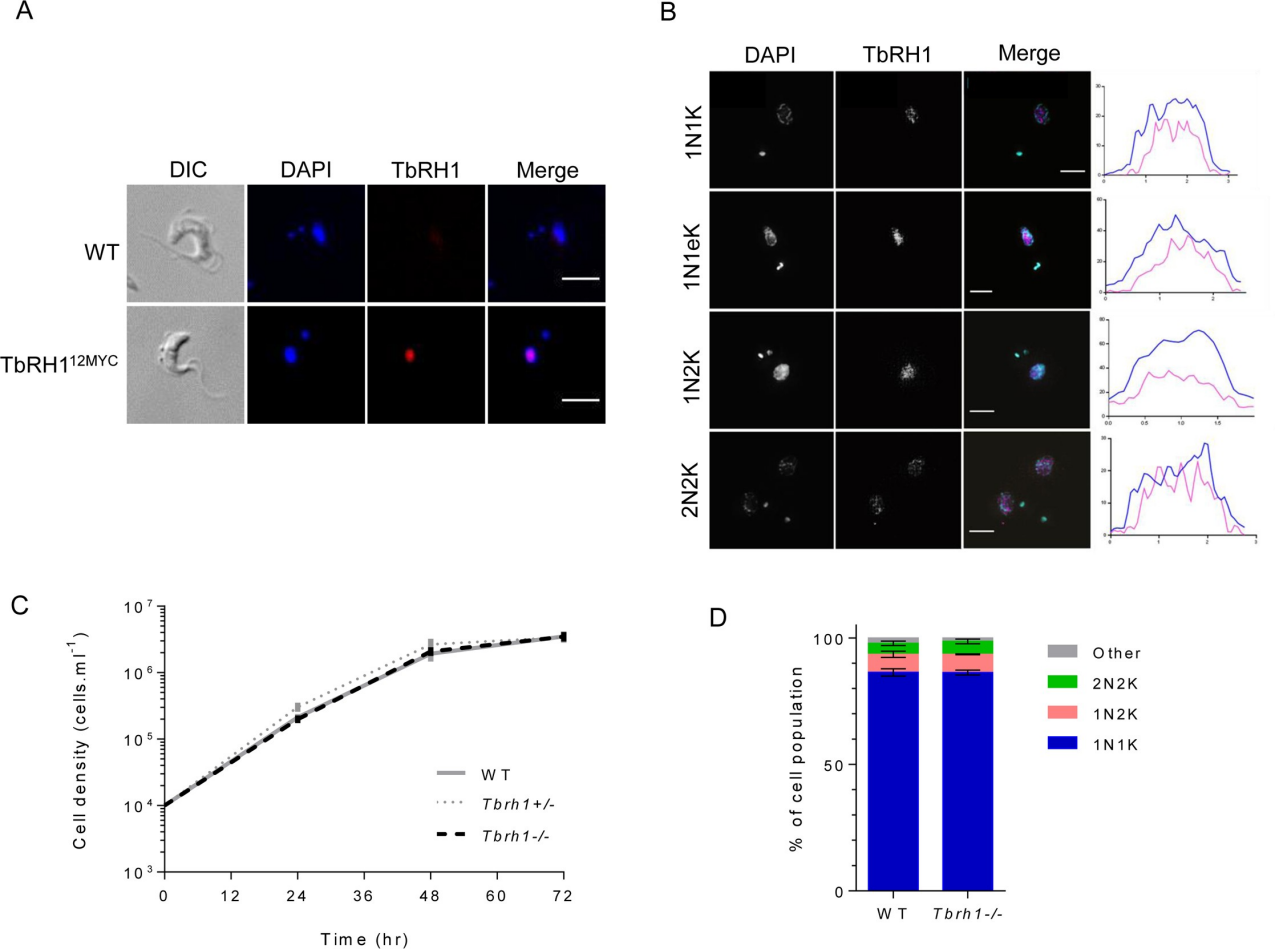
*T*. *brucei* ribonuclease H1 is a non-essential nuclear protein. **A.** Representative immunofluorescence images of *T*. *brucei* cells expressing Ribonuclease H1 (TbRH1) as a fusion with 12 copies of the myc epitope (TbRH1^12myc^); wildtype (WT) cells are shown for comparison. Anti-myc signal is shown in red and DNA is stained with DAPI (blue); merged DAPI and anti-myc signal is also shown, as is the cell outline (by differential interference contrast microscopy; DIC). Scale bars, 5 μm. **B.** Super-resolution structure-illumination imaging of TbRH1 and nuclear DNA colocalisation; TbRH1^12myc^ expressing cells are shown stained with anti-myc antiserum and with DAPI; representative images are shown of different cell cycles stages, and only in the merge of anti-myc (magenta) and DAPI (cyan) images is colour provided. Graphs plot length across the nucleus (x, pixels) versus mean pixel intensity at each position (y, arbitrary units) for DAPI (aqua) and TbRH1 (pink). Scale bars, 5 μm. **C.** Growth of WT cells and *T*. *brucei TbRH1* heterozygous (*TbRH1*+/-) or homozygous *Tbrh1*-/- mutants in culture, with mean population density shown at 24 hr intervals; error bars denote SD from three experiments. **D.** Percentage of the population of WT and *Tbrh1*-/- cells in discernible cell cycle stages, determined by DAPI staining and fluorescent imaging followed by counting the number and shape of nuclear (N) and kinetoplast (K) structures in individual cells: 1N1K, 1N2K and 2N2K and ‘other’ cells that do not conform to these patterns (>200 cells were counted for each cell type).

*T*. *brucei* encodes an RNase H1 homologue (TbRH1) that has been predicted to be nuclear by fusing an N-terminal fragment to GFP [46]. By expressing full-length TbRH1 C-terminally fused to 12 copies of the myc epitope (S1A Fig), from its own locus, we confirmed nuclear localisation (Fig 1A), since anti-myc signal co-localised with the larger (nuclear) DAPI signal and not the smaller (kinetoplast) signal. Expression of TbRH1-myc was constitutive throughout all discernible cell cycle stages of BSF *T*. *brucei* cells and did not display any obvious sub-nuclear localisation (Fig 1B), though increased signal appeared present in cells undergoing nuclear replication (S1B Fig). By integration of *TbRH1* targeting constructs (S2A Fig) we generated heterozygous (+/-) and then homozygous (-/-) TbRH1 mutants (S2B Fig). No growth perturbation (Fig 1C) or alteration in cell cycle stage distribution (Fig 1D) was apparent in the mutants, indicating TbRH1 does not provide essential genome functions (at least in culture), unlike mammalian RNase H1 [47]. However, it should be noted that the analyses described here cannot exclude the potential for secondary mutations during the generation of *Tbrh1-/-* cells, or uncharacterised adaptation following loss of the RNase H enzyme.

### R-loops accumulate throughout VSG transcription sites in the absence of RNase H1

**Fig 2:**
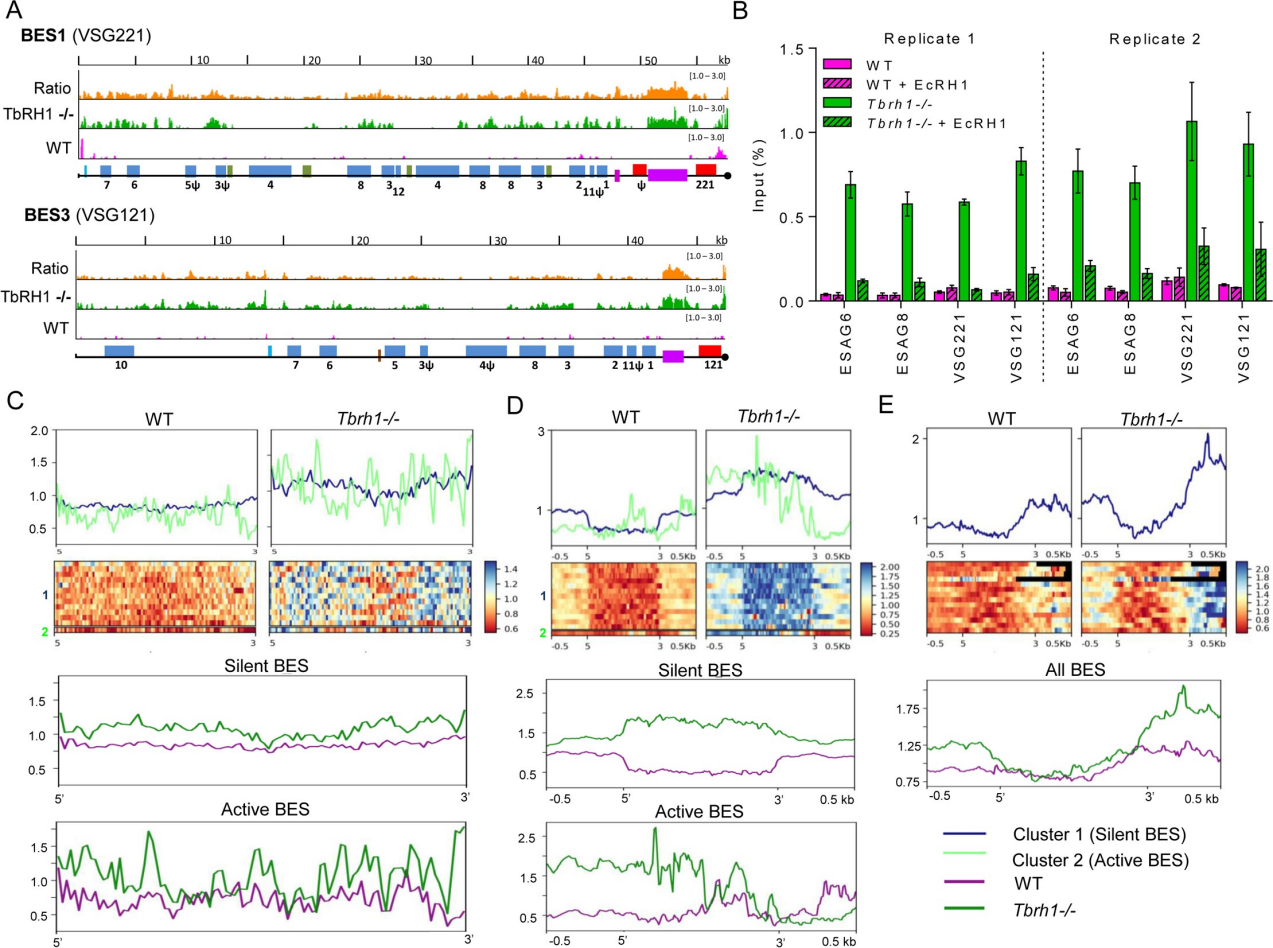
Localisation of R-loops in the Variant Surface Glycoprotein expression sites of *T*. *brucei* before after loss of RNase H1. **A**. Localisation of R-loops by DRIP-seq in wild type (WT) and *T*. *brucei* RNase H1 homozygous mutant (*Tbrh1-/-*) bloodstream form cells. DRIP-seq signal is shown mapped to BES1 (the predominantly active VSG expression site (ES) of WT cells) and BES3 (which is mainly inactive); general structural organisation of the *T*. *brucei* BES is summarised in Fig 5. Pink and green tracks show normalised ratios of read-depth fold-change (1–3 fold) in IP samples relative to input in WT and *Tbrh1*-/- mutants, respectively, while the orange tracks show the ratio of IP enrichment in *Tbrh1-/-* cells compared with WT. Promoters (aqua), *ESAG*s (blue, numbered), 70-bp repeats (purple) and *VSG*s (red) are annotated as boxes; pseudogenes are indicated (ψ), hypothetical genes are shown in green, and the end of the available ES sequence is denoted by a black circle. **B**. DRIP-qPCR, with or without *E*.*coli* RNase H1 (EcRH1) treatment, showing the percentage of PCR amplification in the IP sample relative to input for WT cells (pink) and *Tbrh1*-/- mutants (green); error bars display SEM for at least three technical replicates and data are shown for two biological replicates (1 and 2). **C-E**. DRIP-seq signal fold-change (IP relative to input samples) is shown on the y-axes, plotted as heatmaps and average signal fold-change profiles over ES regions encompassing the *ESAG*s (C), 70-bp repeats (D) and *VSG* (E); for each region, 5’ and 3’ (x-axes) denote the upstream and downstream boundaries, and in some cases -/+ 0.5 kb of flanking sequence is shown. Upper two panels: comparison of WT and *Tbrh1-/-* DRIP-seq signal using K-means clustering, which separated the active (light green, cluster 2) and inactive (dark blue, cluster 1) ES when analysing *ESAG*s and 70 bp repeats, but not *VSG*s. Lower panels: Overlay of WT (purple) and *Tbrh1-/-* (green) DRIP-seq signals in the three ES regions, with the active and silent ES displayed separately for the *ESAG*s and 70 bp repeats.

To ask if TbRH1 targets R-loops within the VSG ES, we performed RNA-DNA immunoprecipitation coupled to next generation sequencing (DRIP-seq) [48] in both wild type (WT) and *Tbrh1-/-* cells, aligning DNA reads to the VSG ES using MapQ filtering [49] to ensure ES-specific mapping of Illumina short reads across regions of homology (Fig 2, S3 Fig). Though a range of strategies to isolate and map R-loops might have been considered [50], DRIP-seq proved valuable in yeast [48, 51, 52] and we therefore deemed it appropriate as a means to provide the first genome-wide view of R-loop distribution in *T*. *brucei* [53]. Here, we have focused on R-loop distribution in the VSG ES. In WT cells there was limited read enrichment across the ES region spanning the promoter to the *VSG*, either in the actively transcribed ES (BES1, containing *VSG221*) or the 13 distinct silent ES (Fig 2A, S3 Fig). Pronounced enrichment was only observed proximal to the ends of the ES, downstream of the *VSG*, which most likely represents TERRA RNA since levels of the signal appeared to increase in *Tbrh1-/-* mutants (Fig 2A and 2E), the opposite of decreased TERRA RNA when TbRH1 is over-expressed [26]. Loss of TbRH1 resulted in DRIP-seq signal throughout all ES, both active and silent (Fig 2A, S3 Fig). To check the mapping, we performed qPCR on DRIP samples (Fig 2B). Enrichment of sequence in the IP relative to input (non-IP) was substantially higher (~10 fold) from *Tbrh1*-/- cells relative to WT for two *ESAG*s (6 and 8), confirming intra-ES R-loops and the increased DRIP-seq signal in the ES of *Tbrh1-/-* mutants compared with WT. The same differential between mutant and WT was also seen with qPCR using primers recognising *VSG221* (BES1, active) or *VSG121* (BES3, inactive), confirming R-loops in both transcribed and untranscribed sites. Finally, on-bead treatment of samples with *E*. *coli* RNase HI prior to DNA recovery clearly reduced the IP enrichment in the *Tbrh1-/-* cells, confirming recovery of RNA-DNA hybrids.

To examine the distribution and abundance of ES R-loops further, we performed K-means clustering of the read alignments from WT and *Tbrh1*-/- mutant DRIP-seq, separating the analysis into three ES components: the *ESAG*-containing region from the promoter to the 70 bp repeats (Fig 2C), the 70 bp repeats (Fig 2D), and the *VSG* plus 500 bp of flanking sequence (Fig 2E). In all components of the ES, as expected, read abundance was greater in the *Tbrh1-/-* mutants than WT. However, the extent and pattern of enrichment was not equivalent in the three components, and nor was it always equivalent in active and silent ES. For the *ESAG* and 70 bp repeat components (Fig 2C and 2D), clustering analysis separated the active ES from all silent ES both in WT and *Tbrh1*-/- cells, suggesting differences dictated by transcription. In contrast, no such separation was seen around the *VSG*, where the active and silent ES could not be distinguished (Fig 2E). Indeed, the level of signal across the *VSG* ORFs was relatively low compared with upstream and, in particular, downstream (presumably telomeric) regions, despite low levels of R-loops being present (as confirmed by VSG DRIP-qPCR; Fig 2B). Enrichment of DRIP-seq signal in *Tbrh1-/-* cells relative to WT extended across the *ESAG*s (Fig 2C) and did not appear to be due to localisation to any specific sequence elements, such as the ORFs or untranslated intergenic regions (Fig 2A, S3 Fig), indicating R-loops became more abundant throughout the region of potential transcription upstream of the 70 bp in the absence of TbRH1. In contrast, the 70 bp repeats were notable for three features (Fig 2D). First, the level of enrichment across the repeats was notably higher in *Tbrh1-/-* mutants relative to WT than in both other components of the ES (~2 fold in the 70 bp repeats, compared with ~1.5 fold elsewhere; see also Fig 2A, S3 Fig). Second, in WT cells the small signal levels in the inactive ES were notably lower across the 70 bp repeats than surrounding sequence, whereas in *Tbrh1-/-* cells signal was greater across the repeats than the flanks. Third, a different pattern was seen in the active ES: in WT cells there was no DRIP-seq signal ‘dip’ within the 70 bp repeats, and in the *Tbrh1-*/- mutants the signal was more enriched at promoter-proximal repeats than telomere-proximal, following the direction of transcription. Taken together, the clustering analysis indicates TbRH1 plays a key role in removing R-loops in the VSG ES, with the 70 bp repeats being a focus for accumulation of the hybrids; furthermore, the distinct features of the mapping within the active ES relative to inactive ES suggest R-loop accumulation and removal by TbRH1 is co-transcriptional in WT cells.

### Loss of RNase H1 results in elevated levels of VSG coat switching

**Fig 3:**
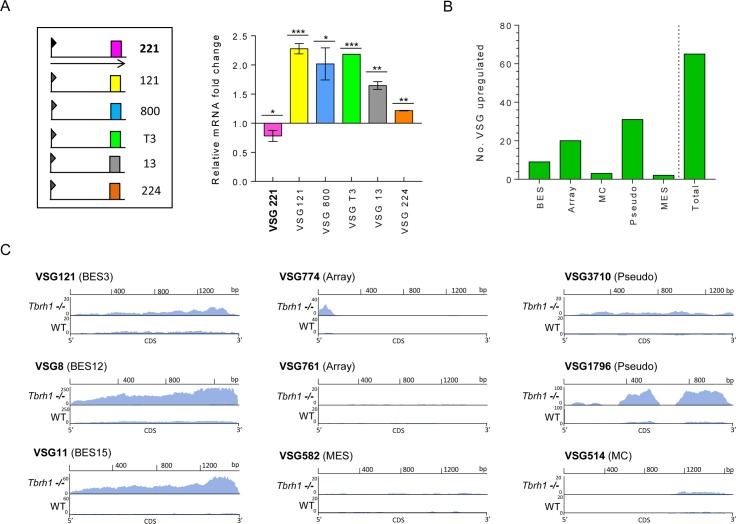
Loss of *T*. *brucei* RNase H1 results in increased transcription of silent VSGs. **A.** The left panel provides a simplified diagram of VSG expression sites (ES) used to generate a protective surface coat in the bloodstream *T*. *brucei* cells used in this study; only telomere-proximal *VSG*s (coloured boxes, numbered) from a selection of ES are shown, and the single ES being actively transcribed (encoding *VSG221*, pink) is denoted by an arrow extending from the promoter (flag). The right panel shows a graph of *VSG* RNA levels (corresponding to the ES diagram, and determined by RT-qPCR) in TbRH1 null mutants (*Tbrh1*-/- cells), plotted as fold-change relative to levels of the cognate *VSG* RNA in wild type cells (WT427 1.2); VSG 221 is in bold to indicate it is in the active ES of wild type cells, and data are presented as mean +/- SD for three independent experiments; * P < 0.05, ** P < 0.005, *** P < 0.0005 (one-way t-test). **B.** A graph depicting the number of VSG genes that display >1.5-fold increase in RNA abundance (determined by RNA-seq, and normalised to gene length and total number of reads) in *Tbrh1*-/- cells relative to WT; the total number is sub-categorised depending on whether the VSGs have been localised to the bloodstream ES (BES), are intact genes in the subtelomeric arrays (array), are in mini-chromosomes (MC), are pseudogenes (pseudo), or are in the ES transcribed in the metacyclic life cycle stage (MES). **C.** Plots of normalised RNA-seq read depth abundance (y-axes) relative to CDS position (x-axes) for a selection of the above VSGs; VSG identity numbers are from [16].

Given that R-loops accumulate within the VSG ES in the absence of TbRH1, we next asked if the increased abundance of the RNA-DNA hybrids is associated with altered VSG expression in *Tbrh1-/-* mutants relative to WT. To test this association, we first performed RT-qPCR to measure RNA levels of a selection of ES *VSG*s (Fig 3A). Five *VSG*s within silent ES in the Lister 427 *T*. *brucei* strain used here [14](Fig 3A) displayed significantly higher RNA abundance in the *Tbrh1-/-* cells relative to WT. In addition, a small reduction (p value 0.0284) in RNA levels was observed for *VSG221*, which is present in the predominantly active VSG ES in WT cells (BES1; Fig 2). To ask if RNA changes are limited to ES VSGs, we performed RNA-seq on RNA from the WT and *Tbrh1-/-* cells and mapped the reads to all available annotated VSGs in the Lister 427 *T*. *brucei* strain [14, 16]. In total, 63 VSGs displayed 1.5 fold or greater numbers of mapped RNA-seq reads in the *Tbrh1-/-* mutants compared with WT (Fig 3B). Amongst these genes were nine bloodstream ES VSGs, of which four showed particularly pronounced read increases, though comparable changes in read depth were not obvious for the associated ESAGs within the ES (see VSG121 in BES3, S4 Fig). Though the RNA-seq and RT-qPCR data do not wholly match (for instance, RT-qPCR predicts some increased VSG224 RNA in the mutants, whereas RNA-seq predicts a decrease; Fig 3 and S4 Fig), it should be noted that the two approaches used independently grown cells. Increased RNA-seq reads in the mutants were also detected for VSGs from all parts of the silent archive, with intact and pseudogenic array VSGs more frequently detected than metacyclic ES or minichromosomal VSGs (Fig 3B and 3C). Taken together, the RT-qPCR and RNA-seq data indicate that loss of RNaseH1 leads to increased levels of transcription from normally silent VSGs, including some not predicted to be resident in VSG ES.

**Fig 4:**
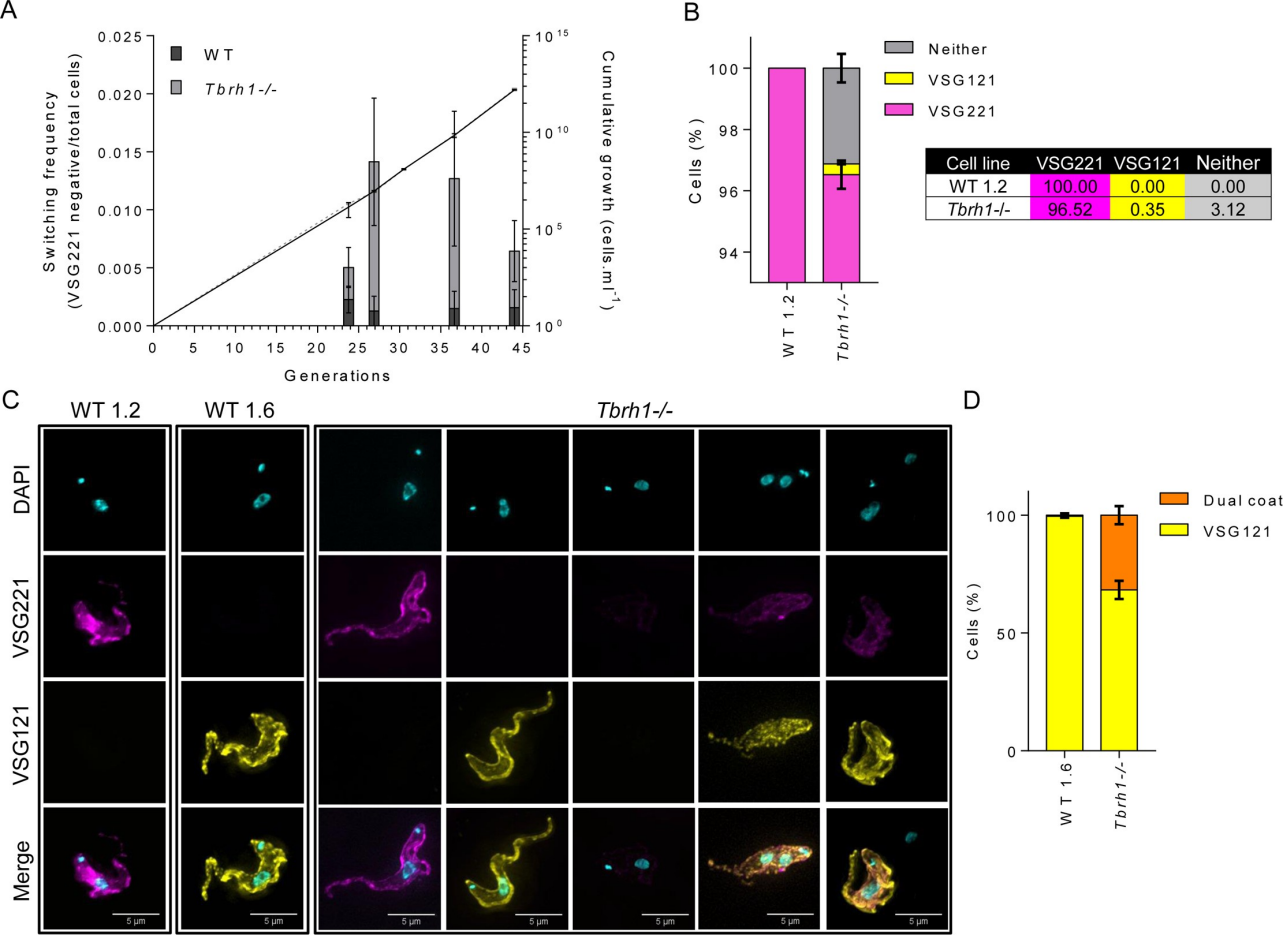
Loss of *T*. *brucei* RNase H1 induces VSG coat switching. **A.** Evaluation of the frequency at which *Tbrh1*-/- and WT cells switch off expression of VSG221 on their cell surface. For each cell type, three VSG221-expressing clones were generated and grown independently, by serial passage in culture, for a number of generations. At multiple time points the number of cells in the populations express or do not express VSG221 was assessed by immunofluorescence with anti-VSG221 antiserum; non-VSG221 expressing WT (black) and *Tbrh1*-/- (grey) cells are shown as a proportion of the total population (data shows average and SD of the three clones, and >200 cells were counted for each clone and at each time point). Cumulative density of WT (solid line) and *Tbrh1*-/- cells (dotted line) over the course of the analysis is shown; values depict average density and SD for the three clones of each cell type at the passages shown. **B** Percentage of WT (WT 1.2) and *Tbrh1*-/- cells expressing VSG221 or VSG121 on their surface, as determined by co-immunofluorescence imaging with anti-VSG221 and VSG121 antiserum. The graph depicts the relative proportions of cells in the population in which only VSG221 (magenta) or VSG121 (yellow) could be detected, as wells as cell with both (orange) or neither (grey) of the two VSG on their surface; >200 cells were analysed for each cell type in each of three replicates (error bars denote SEM). **C.** Co-immunofluorescence imaging of VSG221 and VSG121, providing examples of the surface coat configurations measured in (B); in addition to WT 1.2 cells and *Tbrh1*-/- mutants, an example of a cell is shown from a *T*. *brucei* strain (WT 1.6)[54] that predominantly expresses VSG121 and not VSG221 (Scale bars, 5 μm). **D.** Analysis of WT 1.6 and *Tbrh1*-/- mutant cells that express VSG121 on the cell surface, showing the percentages that simultaneously express VSG221 (orange) or only express VSG121 (yellow); >100 cells were analysed in each of three replicate experiments for each cell type.

To test if these RNA changes extend to the VSG surface coat, we used immunofluorescence on unpermeabilised cells to evaluate the stability of VSG221 expression, since this VSG is normally resident in the predominantly transcribed ES (BES1). To do this, we first examined VSG221 expression over time, comparing the frequency with which three *Tbrh1*-/- and WT clones no longer expressed the protein during prolonged passage (Fig 4A). Despite the absence of immune selection against VSG221 expression, greater numbers of cells without surface VSG221 were seen in the *Tbrh1-/-* cells than in WT at each time point examined, indicating elevated levels of VSG switching throughout growth in culture. Notably, such elevated VSG switching is not associated with changes in population doubling time of the RNaseH1 mutants. To examine this effect in more detail, we next performed co-immunofluorescence (IF) on unpermeabilised WT and *Tbrh1-/-*cells (grown for >45 generations in culture) using antiserum recognising VSG221 (active BES1) or VSG121 (silent BES3). In this WT population, all cells analysed expressed VSG221 (Fig 3B), whereas ~3.5% of the *Tbrh1-/-* mutant cells no longer expressed VSG221 on their surface (Fig 3B and 3C). Most of the cells (~3.1%) that did not react with VSG221 antiserum also did not react with VSG121 antiserum, indicating they expressed a distinct VSG or VSGs on their surface. However, in a small proportion of cells (~0.35%) VSG121 could be detectably expressed, indicating this gene had been activated. To determine if all VSG121-expressing *Tbrh1-/-* cells had switched off VSG221, we looked amongst the VSG121-expressing cells for co-staining with both antisera (Fig 3C and 3D). As a control, immunofluorescence was also performed in a distinct WT strain (i.e. not lacking TbRH1) in which a transcription elongation blockade within BES1 has silenced this ES and predominantly activated BES3 [28, 54, 55], containing *VSG121* (Fig 3C and 3D). Most (~68%) *Tbrh1-/-* cells stained only with anti-VSG121 antiserum, though a minority (~32%) were VSG221-VSG121 double expressers. Taken together, these data indicate that loss of TbRH1 results in an increased frequency at which expression of the active VSG is lost, which mainly reflects complete VSG switching events where the active VSG is no longer detected and expression of a distinct VSG occurs. Loss of mono-allelic control, which results in co-expression of VSGs from the active and at least one previously silent ES, is less common but was observed.

### Loss of RNase H1 results in elevated levels of replication-associated genome damage

**Fig 5:**
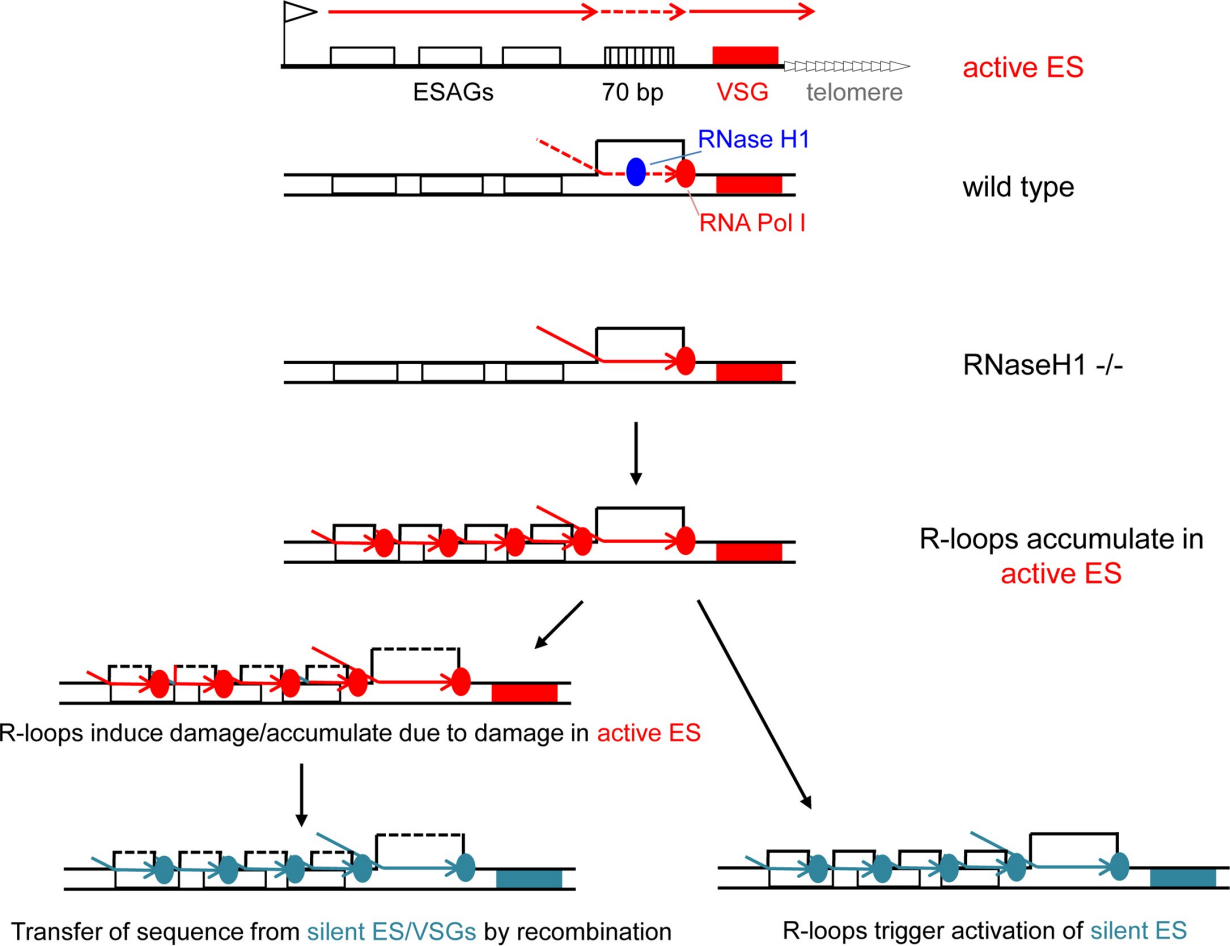
Models of R loop accumulation and resolution in the VSG expression sites of wild type and RNase H1 mutant *T*. *brucei* bloodstream form cells. The topmost diagram summarises the structure of and transcription across (red arrows) the active VSG expression site (ES, black line), including key features: telomere repeats (white arrows), the *VSG* gene (red box), 70 bp repeats (hatched box), *ESAG* genes (white boxes) and the promoter (flag). In the active ES transcription by RNA Polymerase (Pol) I must occur at high levels in order to generate sufficient VSG to form a dense surface coat. Pol I (red circle) passage is slowed when traversing the 70 bp repeats (dotted arrow), leading to the formation of R loops (DNA-RNA hybrid and extruded single strand DNA) that RNase H1 (blue circle) resolves to allow continued transcription. In TbRNaseH1 null mutants (-/-) R loops accumulate in the 70 bp repeats, obstructing transcription and leading to upstream RNA Pol I stalls and the formation of R loops across the length of the ES. Two outcomes of can then occur. In the first, reduced expression of the active ES and VSG is lethal, allowing selection for cells that have activated a distinct ES and VSG (green box). The lack of R-loop resolution by TbRNaseH1 in this site then results in R-loop accumulation and selection for activation of further ES (not shown). In the second outcome, R loops in the active ES lead to, or result from DNA damage, which is then repaired by homologous recombination, resulting in the transfer of sequence (e.g. *VSG*s, *ESAG*s) from the silent archive, including the silent ES, into the active ES.

**Fig 6:**
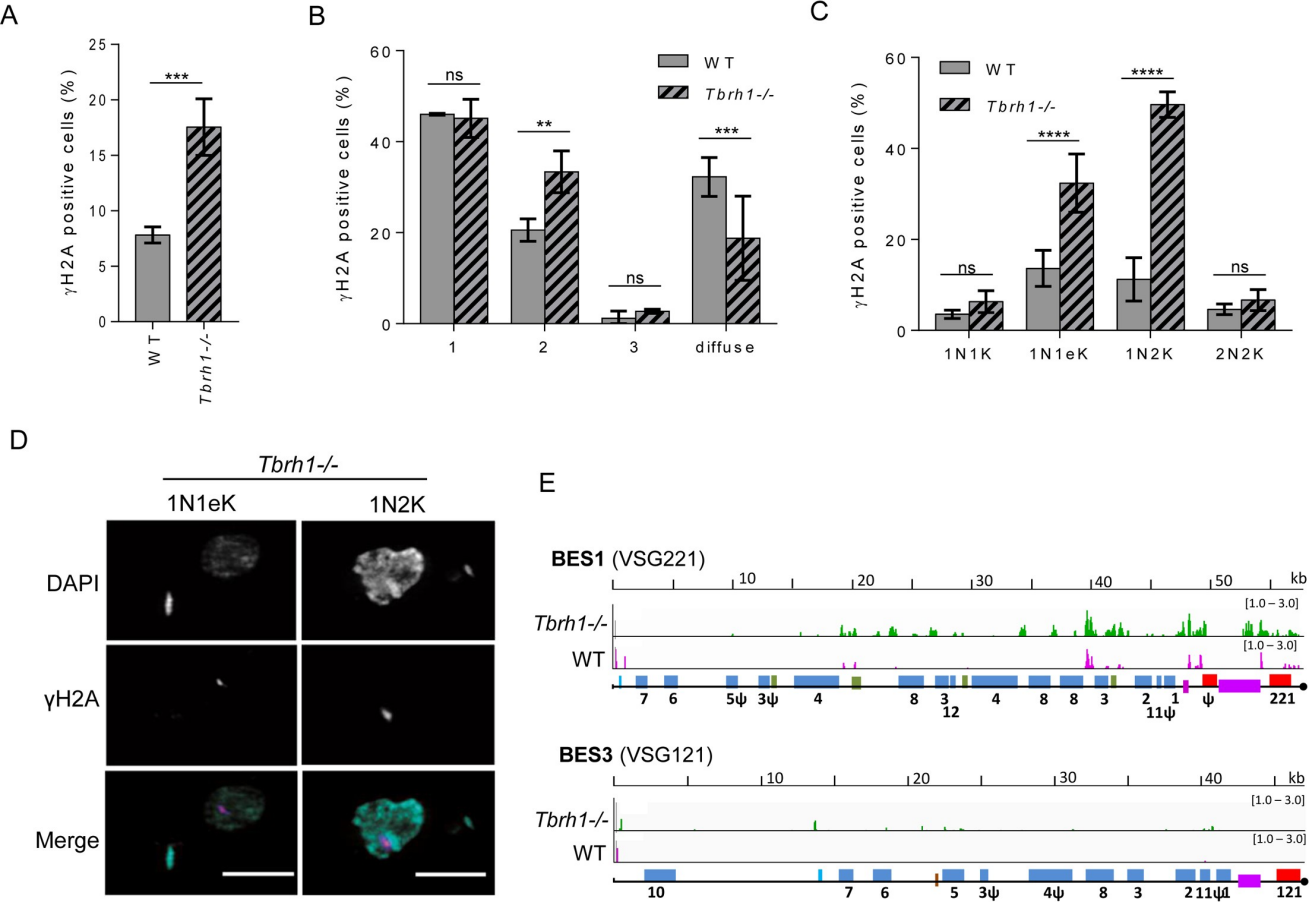
Loss of *T*. *brucei* RNase H1 leads to increased levels of nuclear damage in replicating cells and in the VSG expression site. **A.** Percentage of WT and *Tbrh1*-/- cells (n >200 in each of three replicates) that have detectable nuclear anti-γ-H2A signal. Data shown as mean +/- SD; *** P < 0.001 (unpaired t-test). **B.** Distribution of intra-nuclear γ-H2A signal in WT and *Tbrh1*-/- cells (n >200 in each of three replicates). Data shown as mean +/- SD; ** P < 0.01, *** P < 0.001, ns not significant (two-way ANOVA test). **C.** Percentage of cell cycle stages in WT and *Tbrh1-/-* cells with nuclear anti-γ-H2A signal; cell cycle stages were determined by DAPI staining and fluorescent imaging followed by counting the number and shape of nuclear (N) and kinetoplast (K) structures in individual cells: 1N1K, 1N1elongated K (1N1eK), 1N2K and 2N2K (n ≥50 for each cell cycle stage in three replicates). Data shown and analysed as in C; **** P < 0.0001. **D.** Super-resolution structure-illumination microscopy imaging of anti-γ-H2A signal and co-localisation with DAPI in representative replicating (1N1eK and 1N2K; see below) *Tbrh1-/-* cells; only in the merge of anti-γ-H2A (magenta) and DAPI (cyan) images is colour provided (further examples of mutant and WT cells are provided in S5 Fig). **E.** Localisation of γ-H2A by ChiP-seq in WT and *Tbrh1-/-* cells. ChiP-seq signal is shown mapped to BES1 and BES3, which are represented as in Fig 2. Magenta and green tracks show normalised ratios of read-depth fold-change (1–3 fold) in IP samples relative to input in WT and *Tbrh1*-/- mutants, respectively.

Two models might be considered to explain elevated VSG switching in *Tbrh1*-/- mutants relative to WT cells (Fig 5). In one model, ES R-loop accumulation impedes complete transcription of the active ES, selecting for cells in which a previously silent ES has been transcriptionally activated. Once activated, these newly expressed ES then accumulate R-loops, propagating the DRIP-seq signal across all ES (Fig 2). However, R-loops have also been linked to DNA breaks and rearrangement [39], through impeding DNA replication [56–60], as a result of elevating the levels of transcription-associated breaks [61–63], or because the RNA-DNA hybrids form in response to transcription-associated breaks [64–70]. A second model, therefore, is that increased R-loops in *Tbrh1*-/- cells reflect the accumulation of damage in the ES, leading to recombination-based VSG switching. To try and separate these models, we compared levels of nuclear genome damage in WT and *Tbrh1*-/- cells by assessing expression of Thr130-phosphorylated histone H2A (γ-H2A), which increases in abundance after a range of genotoxic insults [71, 72] and in repair mutants [28]. Western blotting suggested comparable levels of overall γ-H2A in WT and *Tbrh1*-/- cells (S5 Fig). However, IF analysis revealed a ~2.3 fold increase in the number of *Tbrh1*-/- cells with detectable nuclear γ-H2A signal, rising from around 7% in WT (Fig 6A). Super-resolution structure-illumination microscopy revealed that in both WT and *Tbrh1*-/- cells most γ-H2A signal appeared as a single subnuclear focus, though some cells with >1 foci were present (Fig 6B; examples in Fig 6D and S6 Fig). In addition, some cells displayed diffuse staining throughout the nucleus (Fig 6B), suggesting γ-H2A signal may represent various types of damage. DAPI staining of a *T*. *brucei* population provides a means to determine the cell cycle stage of individual cells, since replication and segregation of the nuclear (N) and kinetoplastid (K) genomes occur with different timings [73]. In keeping with previous work [71] more WT cells displayed γ-H2A signal (Fig 6C) when they were undergoing nuclear replication (1N1eK) or were in G2-M phase (1N2K), with reduced numbers of signal-positive cells from the end of M phase (2N2K) through G1 (1N1K). Cell cycle quantification of the *Tbrh1*-/- mutants showed that the increased proportion of cells with γ-H2A signal was nearly entirely accounted for by greater numbers of 1N1eK (~2.4-fold increase) or 1N2K (~3.6-fold) cells with foci relative to WT (Fig 6C), indicating increased accumulation of nuclear damage occurs during replication of the genome in the absence of TbRH1.

### Loss of RNase H1 results in accumulation of DNA damage predominantly in the actively transcribed VSG expression site

To ask if some of the damage detected by microscopy of γ-H2A localises to the VSG ES, we performed ChIP-seq with anti-γ-H2A antiserum in WT and *Tbrh1*-/- cells, mapping the reads to the 14 ES using MapQ filtering (Fig 6E, S7 Fig). In WT cells low levels of γ-H2A ChIP reads were seen in all ES, but the level of enrichment was notably greatest and most widespread in the active ES (BES1, containing VSG221), in particular around the VSG and 70 bp repeat-proximal ESAGs. In the *Tbrh1*-/- cells, γ-H2A ChIP reads were detected at even greater levels in the active ES (BES1), both at the locations detected in WT cells and due to increased reads more proximal to the ES promoter. In contrast, though γ-H2A ChIP reads also increased in the silent ES of *Tbrh1*-/- cells (Fig 6E, S7 Fig), the extent of this change was more modest than was seen in the active ES. These data indicate DNA damage is present in the active ES, in particular proximal to the 70 bp repeats and *VSG*, where distinct assays have suggested the presence of DNA breaks [23–25]. As the extent of γ-H2A signal increases after ablation of TbRH1, this response correlates with the increased abundance of R-loops, which may form preferentially in the active ES.

## Discussion

In this work we reveal that interplay between RNA Pol I transcription and sequence composition of the VSG ES leads to R-loops acted upon by RNase H1, and that loss of the ribonuclease results in increased replication-associated damage, including in the VSG ES, and increased VSG coat switching. These findings suggest VSG transcription and VSG-associated sequences may have evolved to generate R-loops during the expression of the trypanosome’s crucial surface antigen, indicating RNA-DNA hybrids may be harnessed to provide pathogen-specific, discrete functions, such as antigenic variation. These data provide only the second example we are aware of for R-loops providing locus-targeted rearrangement, adding to established roles for R-loops in the initiation of mammalian immunoglobulin class switching [42].

The data we present provide insight into the initiation of antigenic variation in *T*. *brucei* (Fig 5). We suggest that transcription through the VSG ES leads to the formation of R-loops that appear to be rapidly resolved, including by TbRH1, to ensure continued high rates of VSG coat expression [74]. Within the ES the 70 bp repeats appear to be a pronounced site of VSG RNA-DNA hybrid accumulation, given the strongest R-loop ES enrichment is seen in this location in *Tbrh1*-/- mutants. R-loops may form more readily on the 70 bp repeats due to their sequence composition: individual 70 bp repeats show considerable size and sequence variation but are, in part, comprised of (TRR) repeats [15, 75] that can become non-H bonded [76] and promote recombination [77] when transcribed. Increased DRIP-seq signal across the active VSG ES of *Tbrh1*-/- mutants might therefore be most readily explained by R-loops forming initially in the 70 bp repeats and extending back toward the RNA Pol I promoter due to retrograde spreading of the VSG ES transcription blockade. This scenario explains the less pronounced enrichment of R-loops within the telomere-proximal *VSG*s. In addition, it explains the localisation of R-loops both to ES coding and non-coding sequences, which contrasts with the predominant R-loop distribution in multigene transcription units elsewhere in the genome, where R-loops show a very pronounced intragenic localisation [53]. Thus, it is very unlikely that R-loops in the ES are associated with transcription processing, but instead emerge from impaired RNA Pol I movement. However, as we discuss below, R-loops may not only accumulate in RNase H1 mutants because transcription is impeded across the ES, but because of lesions forming within the transcription units.

Our data suggest that R-loop accumulation leads to VSG switching and spreading of R-loops into silent VSG ES by two routes: transcriptional and recombinational. The transcriptional model is consistent with the observation that RNAi-mediated loss of VSG expression from the active ES is lethal [74] and can select for switching [78]. Co-transcriptional R-loop formation might itself lead to reduced transcription of the active VSG ES, or R-loops might form because of lesions generated during ES transcription (see below). Irrespective, once R-loops form in the ES, in particular in the absence of TbRH1, they could provide a blockade to full transcription, causing further R-loops to form in the ES and providing a selection for activation of a new ES. Once a new ES is activated, transcription in this locus then gradually suffers the same R-loop blockade, which is exaggerated by the absence of TbRH1. The model is consistent with the presence of cells expressing both VSG221 and VSG121, since co-expression of at least two VSGs indicates altered transcriptional control. However, such double expressers are relatively rare and the seemingly equivalent accumulation of R-loops in both the active ES and in all silent ES is not simple to reconcile with the continued predominant transcription of VSG221 (BES1) in the *Tbrh1*-/- mutants. Therefore, a second possibility is that R-loops do not merely reflect or cause transcription blockade, but are associated with recombination of silent VSGs (and in some circumstances *ESAG*s from silent ES) into the active ES. An R-loop VSG recombination model is consistent with the greater levels of γ-H2A signal in the active ES and, in this scenario, the relatively similar level of R-loop enrichment in both the active and silent ES could be explained by gene conversion of silent ES sequences into the active ES. However, the lack of evidence for increased RNA spanning the silent ES (such as BES3) in the *Tbrh1-/-* mutants contrasts with increased RNA-seq reads for multiple ES VSGs, and does not readily match the greater density of γ-H2A signal proximal to the 70 bp repeats in the mutants. Instead, the γ-H2A ChIP mapping appears consistent with damage initially arising in the telomere-proximal region of the ES, perhaps due the nature of the repeats (above). If correct, this may explain the RNA-seq data, with telomere-proximal damage leading to the activation not simply (or even predominantly) of silent ES VSGs, but any VSG in the silent archive. In this model, accumulation of R-loops throughout the silent ES may indicate *trans* formation of the RNA-DNA hybrids after their generation in the active site, rather than transcription-associated formation. Irrespective of the precise details, the accumulated data presented here suggests that R-loop-associated recombinational switching predominates over R-loop-associated transcriptional switching. Nonetheless, since we could detect *Tbrh1-/-* cells in which at least two VSGs were expressed on the cell surface, a commonality of R-loops acting during transcription-mediated and recombination-mediated antigenic variation in *T*. *brucei* is possible. In other words, these reactions may not be entirely independent in mechanisms but, instead, share a common initiating event. Such commonality might explain why ablation of the homologous recombination factors RAD51, BRCA2 and RAD51-3 impairs both VSG gene conversion and transcriptional switching [79–81], as well as why induction of DNA damage can elevate levels of silent VSG expression [82].

Precisely how R-loops intersect with *T*. *brucei* VSG switching will need to be explored further, though similarities and differences can be drawn with R-loop involvement in mammalian Ig class switch recombination. In the mammalian IgH locus, R-loops form due to transcription of non-coding RNA upstream of Ig constant region exons [83–85], with evidence for isotype transcription specificity [86]. In *T*. *brucei* each VSG ES is multigenic, with the VSG and ESAGs transcribed from a common upstream RNA Pol I promoter, and no stable intra-ES non-coding RNAs have been described. Thus, it seems likely that R-loops mediating *T*. *brucei* VSG switching are less gene- or exon-specific than those directing mammalian Ig class switching. If so, this difference may explain the mechanistic connection we propose between VSG switching through transcriptional and recombinational routes, whereas class switching is strictly a recombination-catalysed reaction. Nonetheless, our data suggest the 70 bp repeats within the VSG ES may be a focus for R-loop formation, perhaps suggesting some broad similarity with the role of G-rich sequences around Ig exons [87], such as a need to use DNA features to target R-loops. However, in class switch recombination the key role of G-rich sequences appears to be in generating RNA quadruplexes, which recruit the cytidine deaminase enzyme AID [85]; such a role is in keeping with Ig exon expression specificity in B lymphocytes, since G quadruplexes are abundant throughout mammalian genomes but rarely form such structures when transcribed into RNA [88]. 70 bp repeats, in contrast, appear to be nearly exclusively associated with VSGs [89] and no evidence has been documented that they form stable RNAs.

Despite the above potential similarities, execution of Ig class switching and VSG switching by recombination is very different. During Ig class switching RNA quadruplexes target AID to the Ig exons, at which time the enzyme catalyses insertion of uracil residues, leading to DNA double strand breaks (as a result of mismatch and base excision repair) that result in exon deletion through non-homologous end-joining [42]. *T*. *brucei* VSG switching involves homologous recombination [22], and how such a difference in gene rearrangement strategy might emerge from a common R-loop intermediate is unclear. To date, no enzymatic activity, akin to AID, has been described to generate the DNA lesions expected to initiate VSG switching. It is possible that accumulation of R-loops due to ES transcription pausing might alone be enough to generate the increased damage we detect in the active ES. For instance, prolonged negative supercoiling downstream of RNA Pol I might increase the likelihood of R-loop formation and greater exposure of the single-stranded DNA in the RNA-DNA hybrid. Alternatively, R-loops may not be the cause of damage but may form in response to the formation of lesions by another route. Both possibilities are consistent with the detection of putative DSBs within VSG ES [23–25]. Determining whether R-loops cause or respond to DNA breaks remains challenging in any setting [38, 64], since it is clear that chromatin and repair pathways can modulate the damaging effects of R-loops [90–93], while at the same time single- or double-stranded DNA breaks can induce R-loop formation [65, 70]. Key to understanding how R-loops act in VSG switching might be examination of the factors that act on these structures, such as DNA or RNA helicases [94]. In the above scenarios it is assumed that R-loops form during transcription, or that transcription of the ES provides access to a lesion-forming machinery. It is equally possible that R-loops form not simply due to transcription, but impede DNA replication through the active VSG ES and lead to breaks that elicit a VSG switch. This final route would be compatible with the elevated levels of γ-H2A in replicating *Tbrh1*-/- cells, and with previous observations showing the active VSG ES replicates earlier than all the silent ES [28]. In other words, it is possible that ES structure and function has evolved to target replication-transcription clashes to the site of VSG expression to facilitate switching. If so, there may be a parallel with Ig class switching, since R-loops in the IgH locus recruit replisome components and lead to DNA replication [95]. Whether such a putative connection between R-loops, transcription and replication in the VSG ES could recruit repair machinery related to that described in yeast and mammals [90, 96, 97] is unknown.

Currently none of the above scenarios can be ruled out, but the effects of TbRH1 loss on inducing VSG switching appear consistent with observations in other eukaryotes, and with the previously characterised roles of several *T*. *brucei* repair factors. Mutation of *T*. *brucei* RAD51, the key catalytic enzyme of homologous recombination, impairs VSG switching [79]. Intriguingly, yeast Rad51 has recently been shown to promote formation of R-loops and rearrangement at certain loci [98], an effect that is abrogated when factors that promote Rad51 activity are mutated, consistent with the impairment of VSG switching in *T*. *brucei* BRCA2 and RAD51-3 mutants [80, 81]. In both yeast and mammals, loss of the RecQ helicases Sgs2 and BLM, respectively, causes elevated levels of R-loops and locus-specific instability [99], a response that may explain increased VSG switching by gene conversion when RECQ2 (the *T*. *brucei* orthologue) is mutated [28]. Moreover, the R-loop role of Sgs2/BLM has been interpreted as being necessary to tackle replication-transcription clashes, a role that may explain the distinct phenotypes of *T*. *brucei* RECQ2 mutants when acting on DNA double strand breaks and during VSG switching [28]. Finally, mutation of both RNase H enzymes in yeast has been documented to cause elevated levels of DNA damage (detected as RAD52 localisation) at rRNA genes, an effect that is due to RNA Pol I transit and results in gene conversion by break-induced replication [100], a process that has been suggested to mediate VSG switching in the RNA Pol I-transcribed ES [101, 102]. To understand the relationship between R-loops and VSG switching, a number of questions need to be addressed, including: do R-loops generate or follow from ES lesions; what form of DNA lesion results from, or generates the R-loops; how are R-loop-associated lesions signalled to initiate repair; and do increased levels of R-loops in the silent ES indicate movement of the hybrids *in trans* from the active ES? Irrespective of the detailed mechanism, positioning of the 70 bp repeats immediately upstream of the *VSG* appears advantageous, targeting breaks to allow recombination-mediated break repair to access any of the ~1000 *VSG*s outside the VSG ES. Notably, the increased expression of silent VSG RNA in the *Tbrh1*-/- mutants, encompassing the range of genes and loci that comprise the VSG archive, differs from the more limited range of VSG types activated after the controlled generation of a DSB in the active ES [15]. In addition, R-loops that form upstream of the 70 bp repeats in the active VSG ES could drive intra-ES recombination, which is observed frequently [103].

Beyond the proposed mechanistic involvement of R-loops in directing VSG switching, this work reveals wider overlap with emerging roles for RNA in many immune evasion strategies. Antigenic variation in *Neisseria gonorrhoeae* relies upon the expression of a small non-coding RNA, upstream of and antisense to the *pilE* expression site, across a guanine quartet-forming DNA sequence [104], which results in DNA nicks that may elicit recombination [105]. Intriguingly, small RNAs may also be generated from silent *pilS* recombination substrates [106]. Non-coding RNA is widespread in *Plasmodium*, including sense and antisense non-coding RNA (ncRNA) that emanates from the promoter [107] and intron [108] of *var* genes, which mediate antigenic variation, as well as from a *var*-associated GC-rich ncRNA gene family [109]. Modulation of the expression of the ncRNAs, as well as mutating a novel exoribonuclease [107], undermines the transcriptional controls that determine singular *var* gene expression during antigenic variation. Though it has not to date been reported that R-loops form in these settings, as we describe for *T*. *brucei*, and the generation and action of effector transcripts is very likely to be organism-specific, it is notable that both recombination and transcription events during antigenic variation are influenced by RNA, which in at least two cases interacts with DNA. Characterising the factors and reactions that act on the *T*. *brucei* ES R-loops to dictate the dynamics of antigenic variation will reveal how similar or distinct the processes are in the different pathogens.

## Methods

### Molecular and cell biology techniques

All cell lines used were bloodstream form parasites, which were maintained in HMI-9 medium supplemented with 10% (v/v) FBS (Sigma-Aldrich, Missouri, USA) and 1% (v/v) of penicillin-streptomycin solution (Gibco) at 37°C and 5% CO2. C-terminus endogenous tagging of TbRH1 was carried out as previous described [110]. Briefly, the C-terminal 626 bp sequence of the *TbRH1* ORF was PCR-amplified using primers CGACG*AAGCTT*CTGCGGATGACGGTAATG and CGACG*AGATCT*TGTGAATCGCCCTTTGGC and cloned into the pNATx12M plasmid containing 12 copies of the c-myc epitope. The construct was then stably transfected into *T*. *brucei brucei* Lister 427 MITat1.2 cells after digestion with *Pst*I. Heterozygous (-/+) and homozygous (-/-) *Tbrh1* knockout cell lines were generated using two constructs containing cassettes of either blasticidin or neomycin resistance genes between α-β tubulin and actin intergenic regions, flanked by sequences homologous to the 5' and 3' UTRs of *TbRH1*, essentially as described in [28]. Homologous flanking regions were PCR-amplified using the following primers: 5' UTR CGACG*GGATCC*TTGCCTTACCCGTGTTTT and CGACG*TCTAGA*CCTTTTCTTTCCCATGGAC, 3' UTR CGACG*CCCGGG*AGGTGTGTATGGGAATGA and CGACG*CTCGAG*GCACCACCCAGTATAGAAA. Total RNA was extracted using an RNeasy Mini Kit (Qiagen) and reverse transcribed with SuperScript II Reverse Transcriptase (Invitrogen) using random hexamer primers. Power SYBR Green Master Mix (Invitrogen) was used to perform qPCR and fold change was calculated using the 2-ΔΔCT method [111].

### ChIP-seq analysis

Both DRIP and γH2A ChIP sample preparation was performed using a ChIP-IT Enzymatic Express kit (Active Motif). Briefly, ~ 2x10^8^ cells were grown to log phase before fixing in 1% formaldehyde for 5 min whilst shaking at room temperature, before 1 mL of 10X Glycine Buffer was added directly to the cells to stop fixation. Cells were then pelleted, re-suspended in Glycine Stop-Fix Solution and shaken at room temperature for 5 min. Cells were next lysed, according to the manufacturer’s protocol, allowing chromatin to be extracted and digested for 5 min with Enzymatic Shearing Cocktail at 37 ˚C to produce ~200 bp fragments. IP was performed overnight at 4 ˚C with 4.5 ng of S9.6 (Kerafast) or 3 μg of anti-уH2A antibody. For DRIP, on-bead treatment of control DRIP samples was performed as previously described [48]. qPCR was performed directly from DNA recovered from DRIP samples, with or without EcRH1 treatment, using SYBR Select Master Mix (Invitrogen). The amount of DNA in IP samples was expressed as a percentage of input DNA, using CT values first adjusted by the dilution factor of each sample.

Library preparation was performed using a TruSeq ChIP Library Preparation Kit (Illumina) and fragments of 300 bp, including adaptors, were selected with Agencourt AMPure XP (Beckman Coulter). Sequencing was performed with an Illumina NextSeq 500 platform. Reads were trimmed using TrimGalore (https://github.com/FelixKrueger/TrimGalore) under default settings before alignment to the Lister 427 bloodstream VSG expression sites using Bowtie2 [112] in "very-sensitive" mode. Reads with a MapQ value <1 were removed using SAMtools [113], leaving at least 30 million aligned reads per sample. The fold change between input and IP read depth was determined for each sample using the DeepTools bamCompare tool (library size was normalised by read count and fold change was expressed as a ratio) and visualised as tracks with IGV [114]. Normalised ratio files were also used to generate plots and perform kmeans cluster analysis using deepTools computeMatrix, plotProfile and plotHeatmap [115] tools.

### RNA-seq analysis

For RNA-seq analysis, total RNA was extracted using the RNeasy Mini Kit (Qiagen). Poly(A) selection and library preparation was then performed using the TruSeq Stranded Total RNA kit (Illumina) and sequencing of 75 bp paired-end reads was performed using the Illumina NextSeq 500 platform.

RKPM was calculated for each available VSG coding region [16] ignoring duplicate reads. Fold-change in RPKM for each VSG was calculated for *Tbrh1-/-* relative to WT. To ask what transcripts displayed altered levels in the *Tbrh1-/-* mutants relative to WT cells fold in RPKM was determined in RStudio. RNA-seq reads were aligned to the Lister 427 VSG ES [14] and annotated VSGs [16] using HISAT2 [116] in ‘no splice alignment’ mode; reads with a MapQ value <1 were removed using SAMtools, which has been shown to remove >99% of short read alignment to the wrong ES [49]. Read mapping was visualised using Matplotlib and a custom python script.

### Immunofluorescence

VSG immunofluorescence analysis was performed as previously described [18]. Briefly, cell were fixed in 1% formaldehyde (FA) at room temp for 15 min. Cells were then blocked in 50% foetal bovine serum (FBS) for 15 min before primary (α-VSG221, 1:10000; α-VSG121, 1:10000: gift from D. Horn) and secondary (Alexa Fluor 594 goat α-rabbit (Molecular Probes) 1:1000; Alexa Fluor 488 goat anti-rat (Molecular Probes), 1:1000) antibody staining was carried out at room temp for 45 mins in both cases. Cells were then mounted in Fluoromount G with DAPI (Cambridge Bioscience, Southern Biotech). For 12myc-TbRH1 and yH2A staining, cells were first adhered to slides before fixing in 4% FA for 4 min and then quenched in 100 nM glycine. Cells were permeabilised in 0.2% triton-X 100 for 10 min. Blocking was performed for 1 hour with 3% FBS before antibody staining (α-myc Alexa Fluor 488 conjugated (Millipore), 1:500; α-γH2A, 1:1000, and Alexa Fluor 488 goat α-rabbit (Molecular Probes), 1:1000) and mounting in DAPI as described above. For counting purposes cells were imaged using an Axioscope 2 fluorescence microscope (Zeiss) with a 60x objective. Higher resolution of VSG staining was performed with a DeltaVision Core Microscope (Applied Precision), using a 100x 1.4 oil objective (Olympus). Super-resolution structured-illumination imaging of 12myc-RH1 and yH2A signal was performed using an Elyra PS.1 microscope (Carl Zeiss) using a 63x 1.4 objective.

Sequences used in all mapping analyses are available in the European Nucleotide Archive (accession number PRJEB21868).

## Supporting information

S1 Fig Ribonuclease H1 is a nuclear protein in bloodstream form *T. brucei* parasites. **A**. Western blot analysis, using anti-myc antiserum, of a *T*. *brucei* clonal cell expressing C-terminally 12myc epitope-tagged TbRH1 from the endogenous locus in bloodstream form *T*. *brucei*; untagged, wild type (WT) cells are shown for comparison, and the estimated size of TbRH1-12myc is indicated (kDa). **B.** Fluorescence signal intensity (a.u., arbitrary units) of DAPI (cyan) and anti-myc signal (magenta) in TbRH1-12myc expressing cells, separated into different discernible cell cycle stages (determined by number and shape of nuclear (N) and kinetoplast (K) structures seen after DAPI staining: 1N1K, 1N1elongatedK (1N1eK), 1N2K and 2N2K); dots denote intensity of individual cells and the median values (horizontal lines) interquartile range (error bars) are shown. Significance was determined by Kruskal-Wallis non-parametric test: (*) p-value <0.05; (**) p-value < 0.01; (***) p-value < 0.001; (****) p-value <0.0001.
(PDF)

S2 Fig Generation and validation of *TbRH1* null mutants in bloodstream form *T. brucei*. **A.** PCR confirmation of replacement of the *TbRH1* open reading frame (ORF) with selective neomycin (*NEO*) and blasticidin (*BSD*) resistance gene cassettes. The upper two gels show PCR targeting the 3’ end of *NEO* or *BSD* resistance genes, testing linkage of these genes to *TbRH1* flanks after cassette insertion into the *TbRH1* locus. The lowest gel shows PCR of part of the *TbRH1* ORF in wild type cells (WT427), in a *NEO* transformant (Tbrh1+/- neo) and in two *NEO* and *BSD* transformants (Tbrh1 null CL1 and CL2). **B.** RT-qPCR of TbRH1 RNA levels, comparing abundance in WT427, *Tbrh1*+/- cells and in *Tbrh1*-/- null mutants; RNA levels in WT cells (relative to a control RNA) were set at 100% and levels in the mutants are shown as a percentage (error bars show SD from three experiments).
(PDF)

S3 Fig DRIP-seq analysis of all bloodstream VSG expression sites characterised in *T. brucei* strain Lister 427. DRIP-seq was performed with wild type (WT) and *Tbrh1-/-* cells and reads mapped to all ES (BES, numbered as in [14]) not shown in Fig 1. Promoters (aqua), *ESAG*s (blue), 70-bp repeats (purple), *VSG*s (red), non-*ESAG* genes (green) and a drug resistance gene (navy) are annotated as boxes. Pink and green tracks show normalised ratios of read-depth enrichment in IP samples relative to input in WT and *Tbrh1*-/- mutants, respectively, while the orange tracks show the ratio of IP enrichment in *Tbrh1*-/- cells compared with WT.
(PDF)

S4 Fig Bloodstream expression site RNA abundance in the presence and absence of RNaseH1. RNA-seq read depth is shown in WT cells and *Tbrh1-/-* mutants across the length of two VSG ES (BES1 and BES2, containing VSG221 and VSG121, annotated as in Fig 2A); note the read depth scale (y-axes) for each is distinct for the ES regions containing the VSG (red box) and the ESAGs (blue boxes, numbered). For all other ES, RNA-seq read depth (normalised to gene length and total number of reads) is shown only for the VSGs, which are numbered according to [16], with the ES that houses them indicated (see S3 Fig).
(PDF)

S5 Fig Expression levels of γ-H2A in *T. brucei* RNasaeH1 mutants do not change substantially compared with wild type cells. **A.** Western blot of γ-H2A, detected by specific antiserum, in wild type (WT) and *T*. *brucei* RNaseH1 null mutants (*Tbrh1*-/-); antiserum detecting EF1-α provides a loading control. **B.** Relative density of γ-H2A western blot signal, normalised to EF1-α, is compared in WT (normalised to 1.0) and *Tbrh1*-/- cells.
(PDF)

S6 Fig Subnuclear γ-H2A foci in replicating *T. brucei* RNasaeH1 mutants and wild type cells. Super-resolution structure-illumination immunofluorescent imaging of anti-γ-H2A signal and co-localisation with DAPI in a number of replicating (1N1eK and 1N2K) *Tbrh1*-/- and wild type (WT) *T*. *brucei* bloodstream cells; only in the merge of anti-γ-H2A (magenta) and DAPI (cyan) images is colour provided. Scale bars, 5 μm.
(PDF)

S7 Fig γ-H2A localisation in all bloodstream VSG expression sites characterised in *T. brucei* strain Lister 427. ChIP-seq was performed with specific antiserum against γ-H2Ain wild type (WT) and *Tbrh1-/-* cells and Illumina reads mapped to all ES (BES, numbered as in[14]) not shown in Fig 1. Promoters (aqua), *ESAG*s (blue), 70-bp repeats (purple), *VSG*s (red), non-*ESAG* genes (green) and a drug resistance gene (navy) are annotated as boxes. Pink and green tracks show normalised ratios of read-depth enrichment in IP samples relative to input in WT and *Tbrh1*-/- mutants, respectively.
(PDF)

S1 Table Underlying data for graphs. (XLSX)
