# Supplementary figures and images for: Ribonuclease H1-targeted R-loops in surface antigen gene expression sites can direct trypanosome immune evasion
Source: PLoS Genet. 2018 Dec 13;14(12):e1007729. doi: 10.1371/journal.pgen.1007729 (PMC6292569; doi:10.1371/journal.pgen.1007729)

Fig.S1

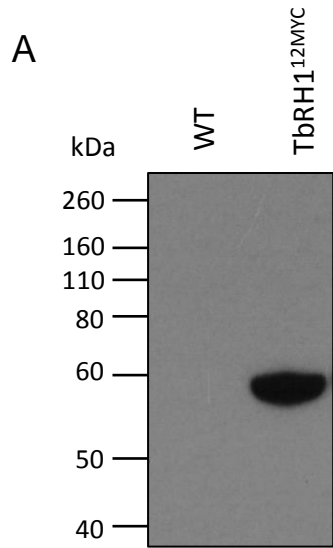

B

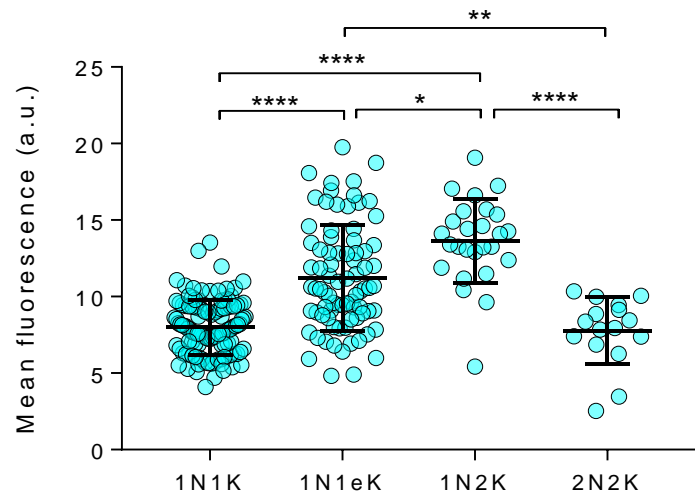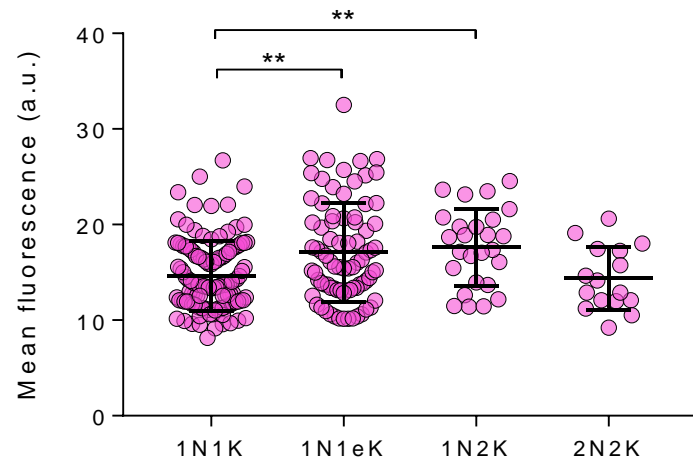

Supplement: S1 Fig — A. Western blot analysis, using anti-myc antiserum, of a T. brucei clonal cell expressing C-terminally 12myc epitope-tagged TbRH1 from the endogenous locus in bloodstream form T. brucei; untagged, wild type (WT) cells are shown for comparison, and the estimated size of TbRH1-12myc is indicated (kDa). B. Fluorescence signal intensity (a.u., arbitrary units) of DAPI (cyan) and anti-myc signal (magenta) in TbRH1-12myc expressing cells, separated into different discernible cell cycle stages (determined by number and shape of nuclear (N) and kinetoplast (K) structures seen after DAPI staining: 1N1K, 1N1elongatedK (1N1eK), 1N2K and 2N2K); dots denote intensity of individual cells and the median values (horizontal lines) interquartile range (error bars) are shown. Significance was determined by Kruskal-Wallis non-parametric test: (*) p-value <0.05; (**) p-value < 0.01; (***) p-value < 0.001; (****) p-value <0.0001. (PDF) [file pgen.1007729.s001.pdf]

Fig.S2

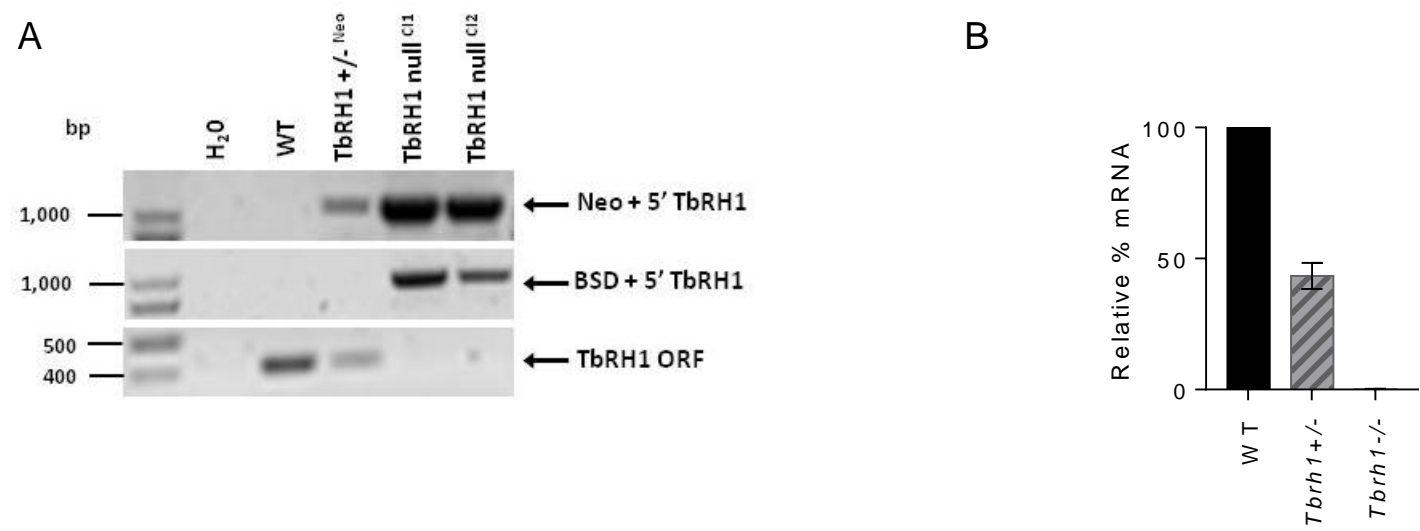

Supplement: S2 Fig — A. PCR confirmation of replacement of the TbRH1 open reading frame (ORF) with selective neomycin (NEO) and blasticidin (BSD) resistance gene cassettes. The upper two gels show PCR targeting the 3’ end of NEO or BSD resistance genes, testing linkage of these genes to TbRH1 flanks after cassette insertion into the TbRH1 locus. The lowest gel shows PCR of part of the TbRH1 ORF in wild type cells (WT427), in a NEO transformant (Tbrh1+/- neo) and in two NEO and BSD transformants (Tbrh1 null CL1 and CL2). B. RT-qPCR of TbRH1 RNA levels, comparing abundance in WT427, Tbrh1+/- cells and in Tbrh1-/- null mutants; RNA levels in WT cells (relative to a control RNA) were set at 100% and levels in the mutants are shown as a percentage (error bars show SD from three experiments). (PDF) [file pgen.1007729.s002.pdf]

Fig.S3

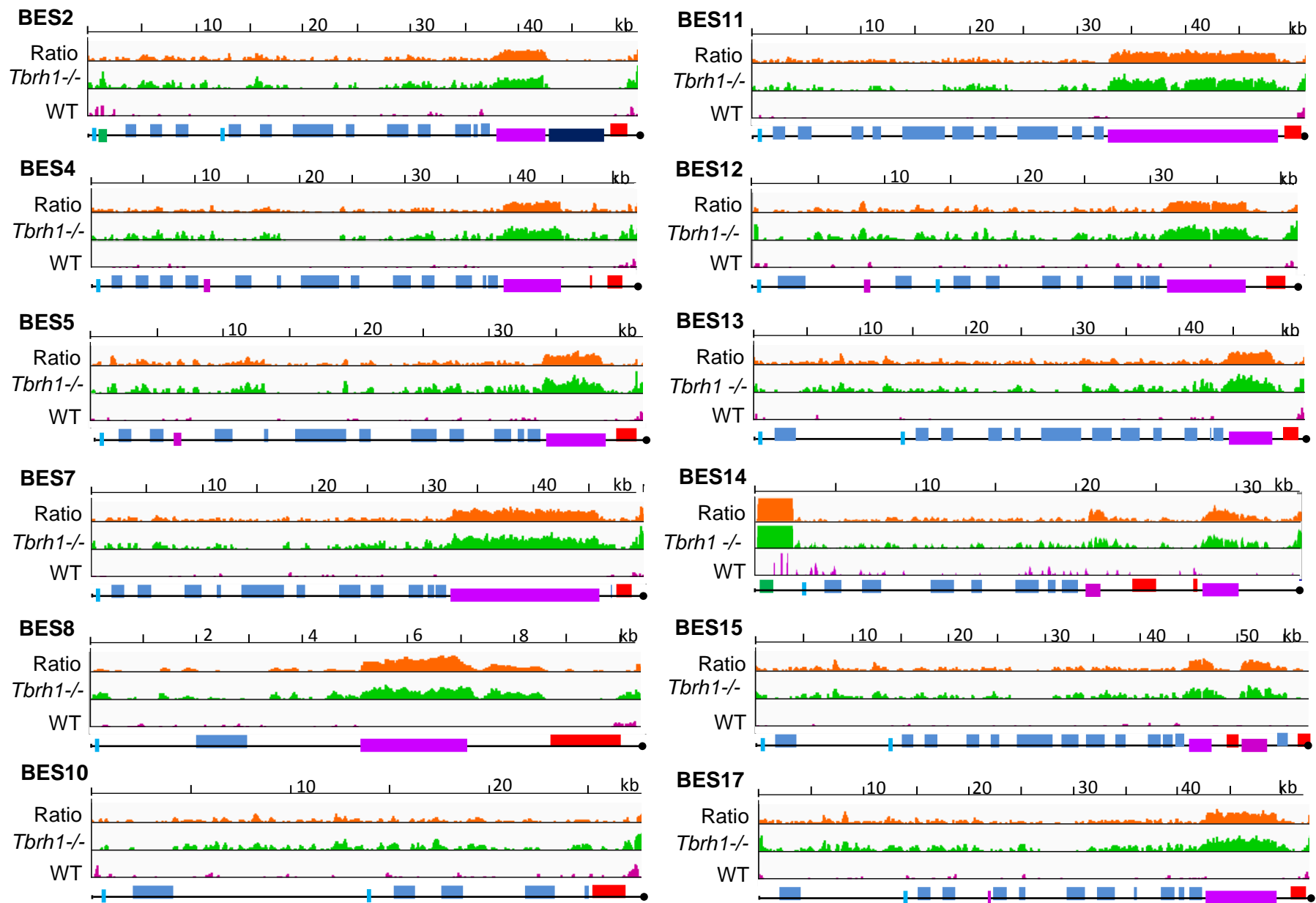

Supplement: S3 Fig — DRIP-seq was performed with wild type (WT) and Tbrh1-/- cells and reads mapped to all ES (BES, numbered as in [14]) not shown in Fig 1. Promoters (aqua), ESAGs (blue), 70-bp repeats (purple), VSGs (red), non-ESAG genes (green) and a drug resistance gene (navy) are annotated as boxes. Pink and green tracks show normalised ratios of read-depth enrichment in IP samples relative to input in WT and Tbrh1-/- mutants, respectively, while the orange tracks show the ratio of IP enrichment in Tbrh1-/- cells compared with WT. (PDF) [file pgen.1007729.s003.pdf]

Fig.S5

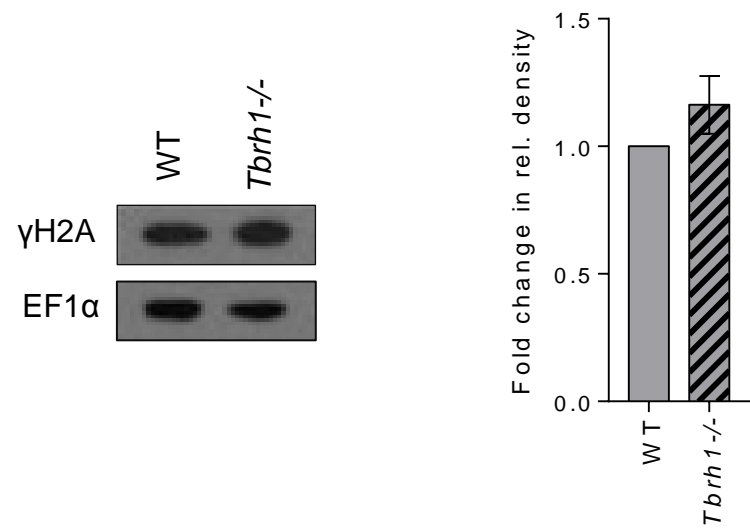

Supplement: S5 Fig — A. Western blot of γ-H2A, detected by specific antiserum, in wild type (WT) and T. brucei RNaseH1 null mutants (Tbrh1-/-); antiserum detecting EF1-α provides a loading control. B. Relative density of γ-H2A western blot signal, normalised to EF1-α, is compared in WT (normalised to 1.0) and Tbrh1-/- cells. (PDF) [file pgen.1007729.s005.pdf]

Fig.S6

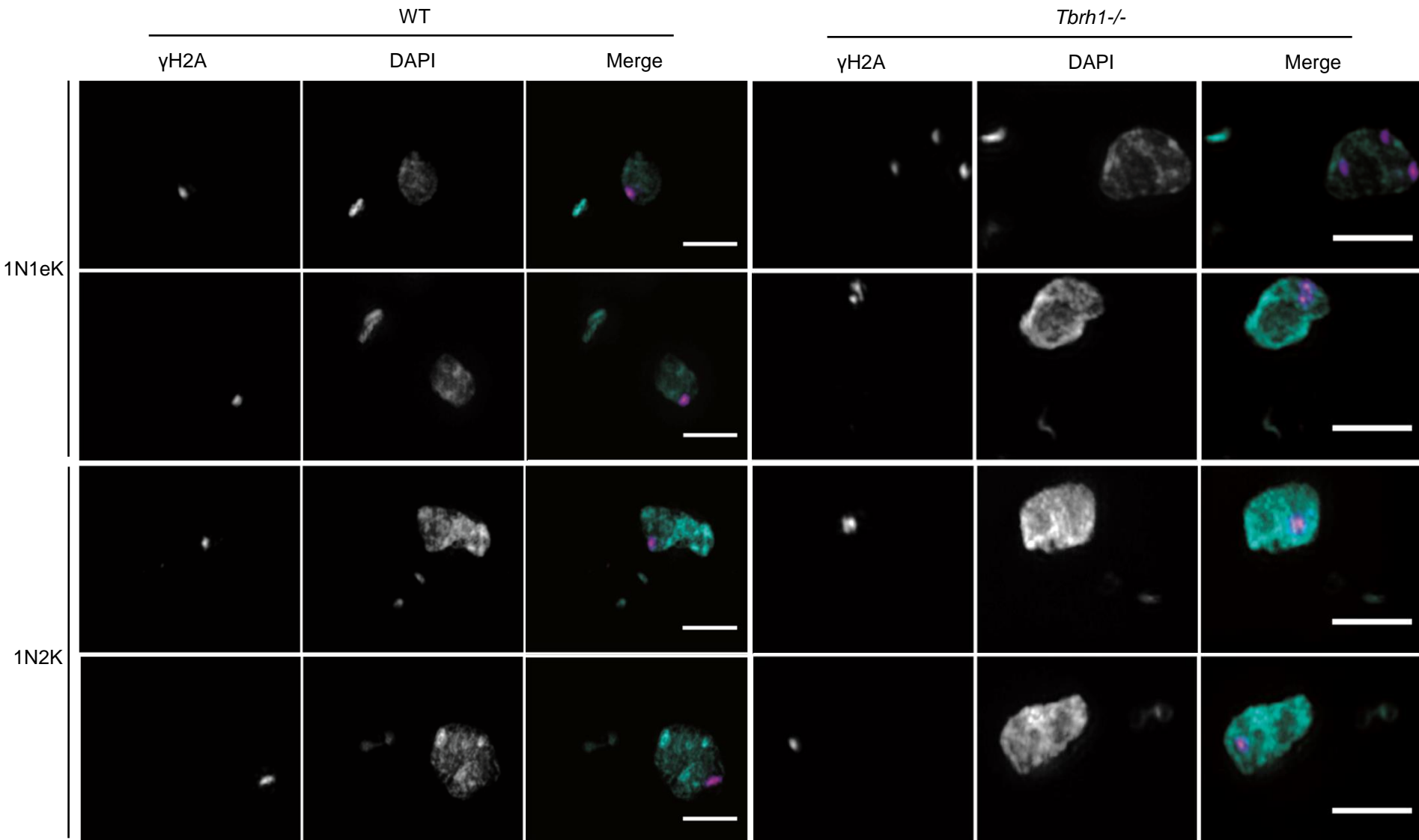

Supplement: S6 Fig — Super-resolution structure-illumination immunofluorescent imaging of anti-γ-H2A signal and co-localisation with DAPI in a number of replicating (1N1eK and 1N2K) Tbrh1-/- and wild type (WT) T. brucei bloodstream cells; only in the merge of anti-γ-H2A (magenta) and DAPI (cyan) images is colour provided. Scale bars, 5 μm. (PDF) [file pgen.1007729.s006.pdf]

Fig.S7

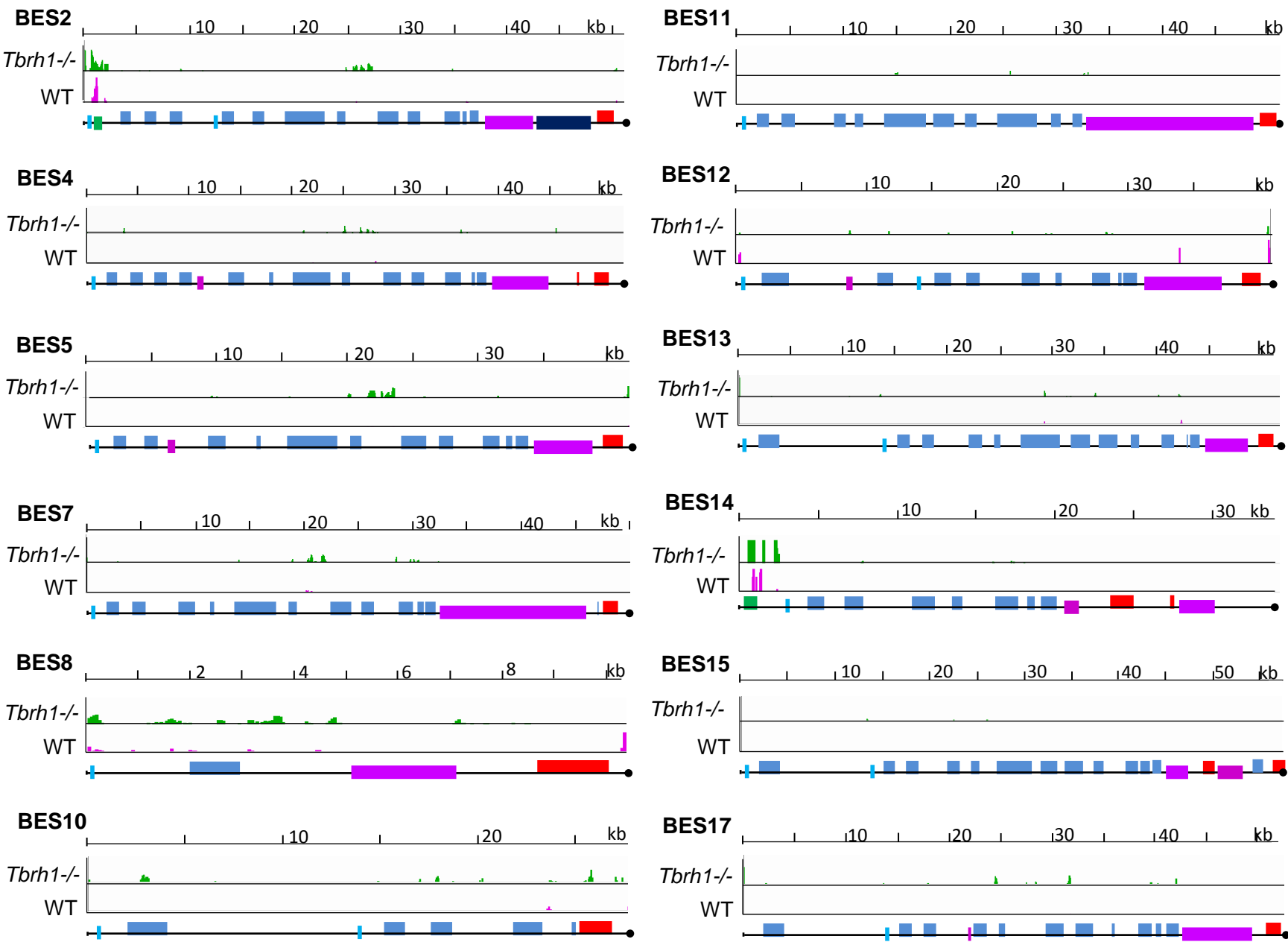

Supplement: S7 Fig — ChIP-seq was performed with specific antiserum against γ-H2Ain wild type (WT) and Tbrh1-/- cells and Illumina reads mapped to all ES (BES, numbered as in[14]) not shown in Fig 1. Promoters (aqua), ESAGs (blue), 70-bp repeats (purple), VSGs (red), non-ESAG genes (green) and a drug resistance gene (navy) are annotated as boxes. Pink and green tracks show normalised ratios of read-depth enrichment in IP samples relative to input in WT and Tbrh1-/- mutants, respectively. (PDF) [file pgen.1007729.s007.pdf]
