# Supplementary material for: Ribonuclease H1-targeted R-loops in surface antigen gene expression sites can direct trypanosome immune evasion
Source: PLoS Genet. 2018 Dec 13;14(12):e1007729. doi: 10.1371/journal.pgen.1007729 (PMC6292569; doi:10.1371/journal.pgen.1007729)
Supplement: S4 Fig — RNA-seq read depth is shown in WT cells and Tbrh1-/- mutants across the length of two VSG ES (BES1 and BES2, containing VSG221 and VSG121, annotated as in Fig 2A); note the read depth scale (y-axes) for each is distinct for the ES regions containing the VSG (red box) and the ESAGs (blue boxes, numbered). For all other ES, RNA-seq read depth (normalised to gene length and total number of reads) is shown only for the VSGs, which are numbered according to [16], with the ES that houses them indicated (see S3 Fig). (PDF) [file pgen.1007729.s004.pdf]

Fig.S4

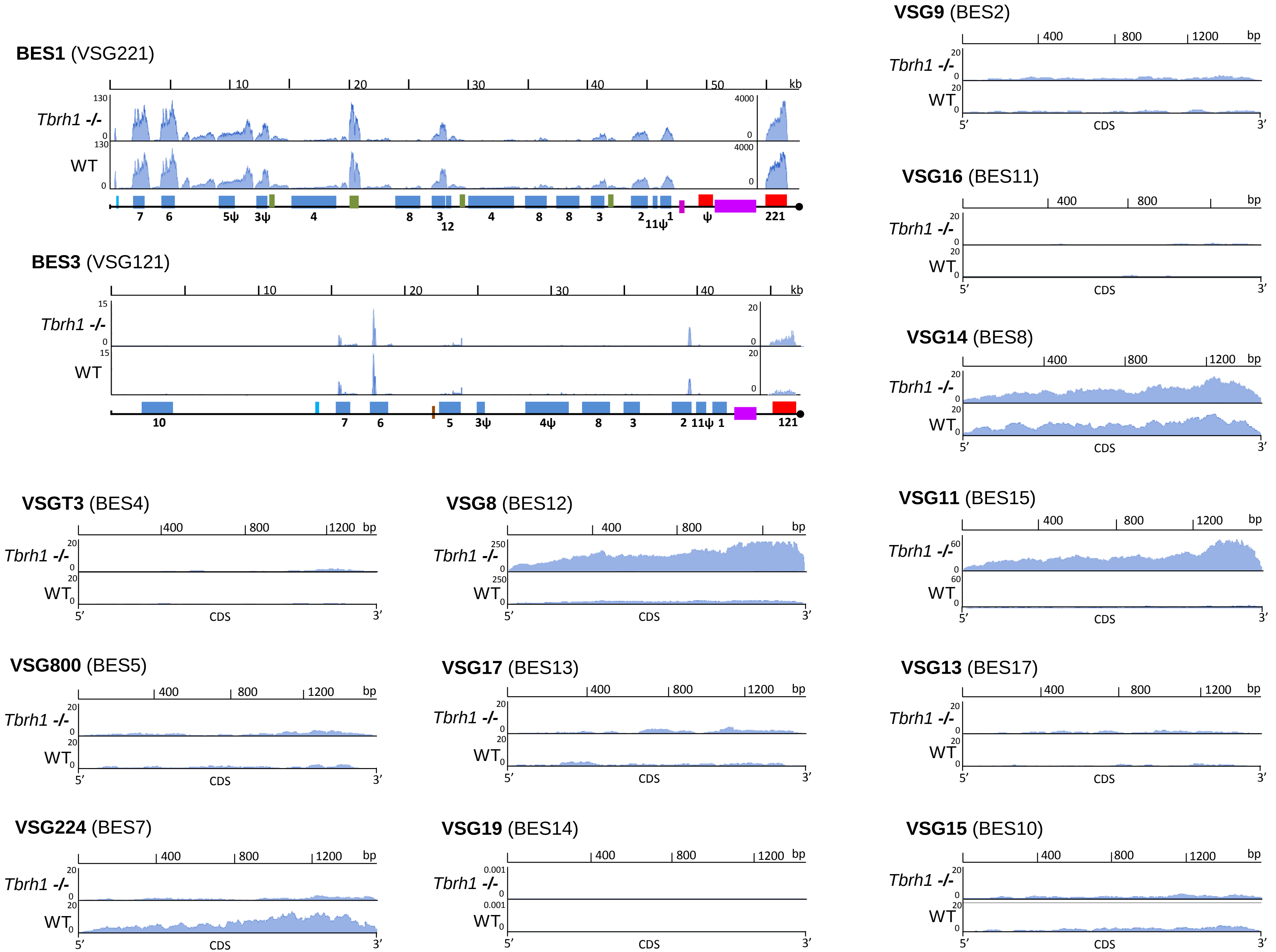

BES1 (VSG221)
BES3 (VSG121)
VSG9 (BES2)
VSG16 (BES11)
VSG14 (BES8)
VSG11 (BES15)
VSGT3 (BES4)
VSG8 (BES12)
VSG800 (BES5)
VSG17 (BES13)
VSG13 (BES17)
VSG224 (BES7)
VSG19 (BES14)
VSG15 (BES10)
Tbrh1 -/-
WT
kb
bp
CDS
5′
3′
